# Safety results of ixekizumab with 1822.2 patient-years of exposure: an integrated analysis of 3 clinical trials in adult patients with psoriatic arthritis

**DOI:** 10.1186/s13075-020-2099-0

**Published:** 2020-01-21

**Authors:** Bernard Combe, Proton Rahman, Hideto Kameda, Juan D. Cañete, Gaia Gallo, Noah Agada, Wen Xu, Mark C. Genovese

**Affiliations:** 1 Department of Rheumatology, Centre Hospitalier Universitaire de Montpellier, University of Montpellier, 34090 Montpellier, France; 2 Memorial University, St. John’s, Newfoundland Canada; 3 Division of Rheumatology, Department of Internal Medicine, Toho University, Tokyo, Japan; 4 Hospital Clinic and Institut d’Investigacions Biomèdiques August Pi i Sunyer (IDIBAPS), Barcelona, Spain; 5 Eli Lilly and Company, Indianapolis, IN USA; 6 Department of Medicine, Stanford University, Stanford, CA USA

**Keywords:** Ixekizumab, Safety, Psoriatic arthritis, Inflammatory bowel disease, Major adverse cardiovascular events

## Abstract

**Background:**

The long-term safety was assessed in patients with psoriatic arthritis who were treated with ixekizumab in three clinical trials (SPIRIT-P1/-P2/-P3).

**Methods:**

Integrated safety data from three trials (controlled and uncontrolled), including two pivotal phase 3, randomized, double-blind clinical trials: SPIRIT-P1 and SPIRIT-P2, were assessed. Safety data were integrated from the all ixekizumab exposure safety population (defined as all patients receiving ≥ 1 dose of ixekizumab). We report exposure-adjusted incidence rates (IRs) per 100 patient-years (PY) at 1-year intervals up to 3 years for adverse events.

**Results:**

Total exposure to IXE reached 1822.2 PY (1118 patients). The IRs/100 PY for the following treatment discontinuations were as follows: adverse events (5.3); serious infections (1.3); injection-site reactions (12.7); infections (34.2); and deaths (0.3). The IRs for treatment-emergent adverse events decreased or remained stable over time, the most common being upper respiratory tract infection, nasopharyngitis, and injection-site reactions. The IRs for serious adverse events and serious infections remained stable over time, whereas for injection-site reactions and general infections, IRs decreased with longer ixekizumab exposure. Opportunistic infections were limited to oral and esophageal *candida* and localized herpes zoster. No suicide or self-injury-related behaviors were reported. The IRs/100 PY for safety topics of special interest included inflammatory bowel disease (adjudicated; 0.1), depression (1.6), malignancies (0.7), and major adverse cardiovascular events (0.6).

**Conclusions:**

The findings of this integrated safety analysis in patients with psoriatic arthritis are consistent with the known safety profile of ixekizumab. No unexpected safety signals were observed with ixekizumab treatment in patients with psoriatic arthritis.

**Trial registration:**

SPIRIT-P1 (NCT01695239; Registered August 08, 2012), SPIRIT-P2 (NCT02349295; September 23, 2014), and SPIRIT-P3 (NCT02584855; August 04, 2015).

## Background

Psoriatic arthritis (PsA) is a chronic, inflammatory disease, which is characterized by peripheral arthritis, axial disease, enthesitis, dactylitis, and skin and nail manifestations [1]. Ixekizumab (IXE) is a high-affinity monoclonal antibody that selectively targets interleukin 17A (IL-17A) [2]. The United States Food and Drug Administration has approved IXE for the treatment of psoriasis, psoriatic arthritis, and axial spondyloarthritis [3]. Due to the chronic nature of this disease, long-term safety data on IXE are critical.

In clinical the SPIRIT-P1 trial, IXE was superior to placebo (PBO) in improving several measures including disease activity, radiographic disease progression, physical function, and patient-reported quality of life in biologic-naïve patients with active PsA [4]. In clinical trial SPIRIT-P2, IXE improved the signs and symptoms of patients with active PsA (inadequate responders to tumor necrosis factor [TNF] inhibitor) along with a safety profile consistent with previous studies involving both PsA and psoriasis [5, 6].

A previously published integrated analysis paper by Mease et al., from three clinical trials showed no unexpected safety signals with IXE treatment up to week 96 [7]. We report the results of integrated analysis that evaluated long-term safety and tolerability of up to 3 years of exposure to IXE using data from three clinical trials for 1822.2 patient-years (PY) in patients with active PsA.

## Methods

### Patients and study design

**Fig. 1:**
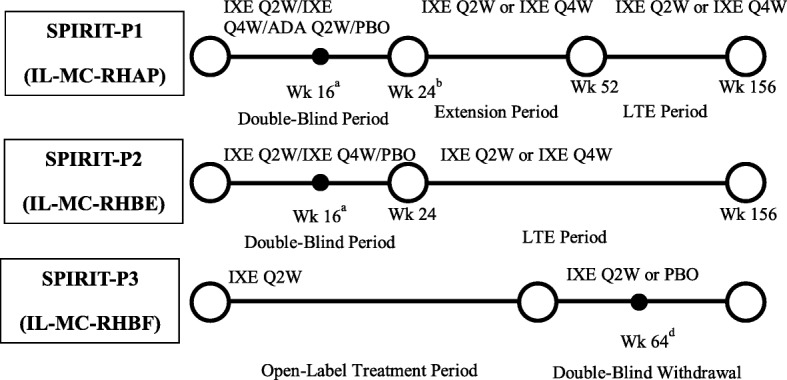
Study design. All patients treated with IXE had a loading dose of 160 mg at week 0. ADA dose was 40 mg Q2W unless stated otherwise. ^a^Patients determined to be inadequate responders by blinded criteria given adjustments to their background/existing therapy. Inadequate responders in the non-IXE groups randomized to IXE Q2W or IXE Q4W with washout for ADA inadequate responders. ^b^Responders in ADA or PBO groups re-randomized to either IXE Q2W or IXE Q4W. ^c^Patients randomized to IXE Q2W or PBO if they met the randomized withdrawal (RW) criteria (i.e., those who met Coates criteria for MDA for ≥ 3 consecutive months across ≥ 4 consecutive visits) at week 36 or later up to week 64. ^d^Patients who had not met RW criteria at week 64 were given IXE Q2W; patients who relapsed (no longer met MDA criteria) during the double-blind withdrawal period were switched to, or continued, IXE Q2W. ADA Q2W: 40 mg of adalimumab every 2 weeks; IXE Q2W: 80 mg of ixekizumab every 2 weeks; IXE Q4W: 80 mg of ixekizumab every 4 weeks; LTE: long-term extension; MDA: minimal disease activity; PBO: placebo; Wk: week

The present report includes integrated safety analysis data derived from SPIRIT-P1 [4], SPIRIT-P2 [5], and SPIRIT-P3 (Fig. 1). The analysis used data from the All-IXE Exposure-Safety Population, defined as all patients with PsA receiving ≥ 1 dose of IXE. This database includes data from all study periods of SPIRIT-P1 and SPIRIT-P2, along with the open-label period of SPIRIT-P3. The results presented here are from a database lock in March 2018 of these three clinical trials.

Clinical trials SPIRIT-P1 and SPIRIT-P2 are phase 3 randomized, double-blind, PBO-controlled, parallel-group trials involving patients with active PsA [4, 5]. Patients were randomized to subcutaneous injections of PBO, adalimumab 40 mg (ADA), IXE 80 mg once every 2 weeks (IXE Q2W), or IXE 80 mg once every 4 weeks (IXE Q4W). Both IXE regimens included a 160-mg starting dose. Patients who received PBO and ADA were re-randomized to either IXE Q2W or IXE Q4W for the open-label extension period (weeks 24–156); patients who initially received IXE remained on their original dose. Both trials have similar study designs, except SPIRIT-P1 patients are biologic naive whereas SPIRIT-P2 patients are conventional (c) disease-modifying antirheumatic drugs (DMARDs) and biologic (b) DMARDs experienced. SPIRIT-P1 included assessments of radiographic progression and used ADA as an active control. The primary efficacy and safety analyses of both trials are published [4, 5]. SPIRIT-P3 is a phase 3 study with an open-label period (weeks 0–36) followed by a randomized double-blind withdrawal period from week 36 to week 104, examining the effect of single-arm IXE Q2W in patients with active PsA who are cDMARD-inadequate responders and bDMARD naive.

All studies included in this analysis were compliant with ethical guidelines including the Declaration of Helsinki and other relevant laws and regulations. The study protocols were approved by each site’s ethical review committee/institutional review board, and all patients provided written informed consent.

### Safety evaluations

Adverse events (AEs) were classified based upon the Medical Dictionary for Regulatory Activities (MedDRA) versions 19.0 and 19.1. A treatment-emergent AE (TEAE) was defined as an event that first occurred or worsened in severity from baseline until, or prior to, the last visit within the treatment period, and which did not necessarily have a causal relation with the study drug.

Prespecified safety topics of special interest included serious infections (SIs), injection-site reactions (ISRs), allergic reaction/hypersensitivity, opportunistic infections (including candidiasis), major adverse cardiovascular events (MACEs), malignancies (excluding non-melanoma skin cancer [NMSC]), tuberculosis (TB), depression, and suicidality. Each adjudicator reviewed suspected inflammatory bowel disease (IBD) cases and reported their findings as definite, probable, or possible using the EPIMAD registry methodology for diagnosis of IBD cases [8]. Only patients with definite or probable Crohn’s disease (CD) or ulcerative colitis (UC) were classified as having IBD. MACEs were adjudicated by a Clinical Events Committee (CEC).

TB screening was performed at week 52 and annually in all patients per the protocol in patients with no history of TB. In SPIRIT-P1, patients were screened for latent TB infection and were required to be negative or to complete 4 weeks of treatment before enrolment. Patients who tested positive were discontinued. In SPIRIT-P2 or SPIRIT-P3, patients continued if active TB were excluded and if they received a full course of treatment for latent TB with no evidence of hepatotoxicity.

### Statistical methods

Overall exposure of IXE was summarized in total PY. This was calculated as follows: PY = sum of duration of exposure in days (for all patients in treatment group)/365.25. TEAEs were summarized by frequencies and exposure-adjusted incidence rates (IRs). IRs per 100 PY were calculated by dividing the total number of patients experiencing the TEAE for the events of interest by the sum of all patients’ time (in 100 years) of exposure during the treatment period. The entire exposure time during the treatment period was used. Frequencies and exposure-adjusted IRs of AEs over time by 1-year time intervals through 156 weeks (3 years) were summarized. The patients who had multiple events across the yearly intervals were counted once in each yearly interval.

## Results

**Fig. 2:**
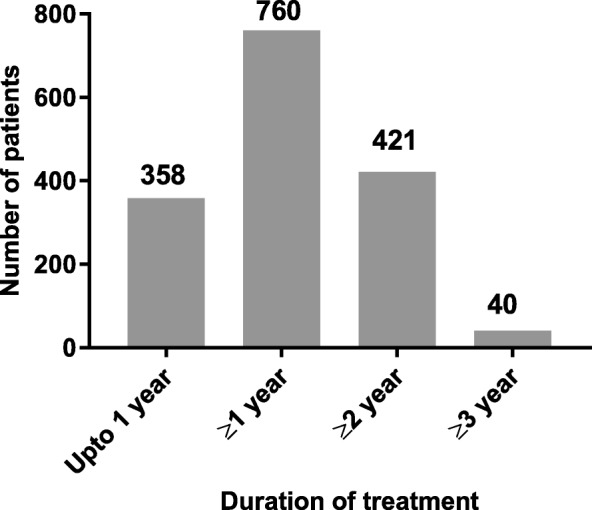
Number of patients by treatment duration. The number of patients exposed to ixekizumab over a period of 3 years. Total *N* = 1118; total exposure = 1822.2 patient-years

A total of 1118 patients who received IXE from 3 studies were included and accounted for 1822.2 PY of exposure (median exposure was 645 days ranging from 8 to 1219 days). The number of patients exposed to study drug over a period of 3 years is shown in Fig. 2. For the pooled population with PsA, the mean age was 49.5 years and 53.8% were female. The mean (SD) duration of the PsA symptoms was 9.71 (8.7) (Table 1).

**Table 1:** Demographic and baseline characteristics (All PsA ixekizumab-exposure safety population)

Characteristics	All-IXE treatment periods(*N* = 1118)
Age, years, mean (SD)	49.5 (11.9)
Sex, *n* (%)	
Male	517 (46.2)
Female	601 (53.8)
Race, *n* (%)	
White	1056 (94.5)
Asian	39 (3.5)
American Indian or Alaska Native	9 (0.8)
Multiple	8 (0.7)
Black or African American	4 (0.4)
Native Hawaiian or other Pacific Islander	1 (0.1)
Weight, kg, mean (SD)	86.31 (20.4)
BMI, kg/m^2^, mean (SD)	29.95 (6.9)
Previous PsA systemic therapy^a^, *n* (%)	
No prior treatment	218 (19.5)
Non-biologic only	562 (50.3)
Biologic only	71 (6.4)
Biologic and non-biologic	267 (23.9)
Duration of PsA symptoms in years, mean (SD)	9.71 (8.7)

All-IXE treatment period defined as all patients who received ≥ 1 dose of IXE

^a^Systemic therapy includes biologic (such as anti-TNF inhibitors) and non-biologic (such as cDMARDs, NSAIDs, and corticosteroids) medications that were used prior to the study entry

*bDMARDs* biologic disease-modifying antirheumatic drugs, *BMI* body mass index, *cDMARDs* conventional disease-modifying antirheumatic drugs, *IXE* ixekizumab, *N* population size, *n* number in each group, *NSAIDs* non-steroidal anti-inflammatory drugs, *PsA* psoriatic arthritis, *SD* standard deviation

The *n* (IRs/100 PY) for TEAEs at years 1, 2, and 3 were 844 (89.3/100 PY), 465 (72.5/100 PY), and 170 (72.4/100 PY), respectively. The most common TEAEs (*n* [IRs/100 PY]) were upper respiratory tract infection (161 [8.8/100 PY]), nasopharyngitis (150 [8.2/100 PY]), and ISR (142 [7.8/100 PY]) (Table 2).

**Table 2:** Summary of most commonly reported adverse events (incidence rates per 100 PY)

Event type	Double-blind period (weeks 0–24)			All-IXE treatment periods (*N* = 1822.2) IR (95% CI)
	Placebo *N* = 224 *n* (IR)	IXE80Q4W (*N* = 229) *n* (IR)	IXE80Q2W (*N* = 225) *n* (IR)	
Patients with ≥ 1 TEAE	127 (148.2)	153 (155.6)	156 (163.4)	50.0 (46.9, 53.4)
Mild^a^	60 (70.0)	91 (92.6)	81 (84.8)	20.4 (18.4, 22.5)
Moderate^a^	63 (73.5)	54 (54.9)	61 (63.9)	24.3 (22.1, 26.6)
Severe^a^	4 (4.7)	8 (8.1)	14 (14.7)	5.4 (4.5, 6.6)
Patients discontinuing from study drug due to AEs	8 (9.3)	7 (7.1)	12 (12.6)	5.3 (4.3, 6.4)
Patients with ≥ 1 SAEs	6 (7.0)	9 (9.2)	11 (11.5)	6.4 (5.3, 7.6)
Deaths	0 (0)	0 (0)	0 (0)	0.3 (0.1, 0.7)
Patients with ≥ 1 most frequent TEAEs (preferred term)				
Upper respiratory tract infection	16 (18.7)	16 (16.3)	15 (15.7)	8.8 (7.6, 10.3)
Nasopharyngitis	9 (10.5)	15 (15.3)	7 (7.3)	8.2 (7.0, 9.7)
Injection-site reaction	1 (1.2)	22 (22.4)	32 (33.5)	7.8 (6.6, 9.2)
Bronchitis	7 (8.2)	4 (4.1)	7 (7.3)	4.4 (3.6, 5.5)
Sinusitis	5 (5.8)	9 (9.2)	6 (6.3)	3.7 (2.9, 4.7)
Urinary tract infection	5 (5.8)	8 (8.1)	4 (4.2)	3.2 (2.5, 4.1)
Injection-site erythema	0 (0.0)	9 (9.2)	17 (17.8)	2.9 (2.2, 3.7)
Patients with ≥ 1 AESIs				
Cytopenias	2 (2.3)	2 (2.0)	4 (4.2)	2.5 (1.9, 3.4)
Hepatic	10 (11.7)	7 (7.1)	11 (11.5)	4.9 (4.0, 6.0)
Infection	62 (72.3)	77 (78.3)	72 (75.4)	34.2 (31.6, 37.0)
Serious infections	0 (0)	1 (1.0)	5 (5.2)	1.3 (0.8, 1.9)
Candida infections	1 (1.2)	4 (4.1)	8 (8.4)	2.1 (1.6, 2.9)
Esophageal candidiasis	0 (0)	0 (0)	1 (1.0)	0.1 (0.0, 0.4)
Active tuberculosis	0 (0)	0 (0)	0 (0)	0 (0.0, 0.0)
Latent tuberculosis	0 (0)	0 (0)	0 (0)	0.7 (0.4, 1.2)
Injection-site reactions	10 (11.7)	40 (40.7)	57 (59.7)	12.7 (11.2, 14.5)
Allergic reactions/hypersensitivities	4 (4.7)	10 (10.2)	14 (14.7)	4.8 (3.9, 6.0)
Confirmed cerebro-cardiovascular events	2 (2.3)	0 (0)	0 (0)	1.2 (0.8, 1.8)
Confirmed MACE events	0 (0)	0 (0)	0 (0)	0.6 (0.3, 1.1)
Malignancies	0 (0)	2 (2.0)	0 (0)	0.7 (0.4, 1.2)
Depression	3 (3.5)	4 (4.1)	4 (4.2)	1.6 (1.2, 2.4)
Adjudicated inflammatory bowel disease (narrow and broad terms)	0 (0)	0 (0)	0 (0)	0.1 (0.0, 0.4)^b^
Adjudicated Crohn’s disease	0 (0)	0 (0)	0 (0)	0.1 (0.0, 0.4)
Adjudicated ulcerative colitis	0 (0)	0 (0)	0 (0)	0.1 (0.0, 0.4)

All-IXE treatment period defined as all patients who received ≥ 1 dose of IXE

^a^Patients with multiple occurrences of the same event are counted under the highest severity

AEs are listed according to the preferred term in MedDRA, and AEs occurred in ≥ 3.0% of the patients in the combined (total) ixekizumab group

^b^The data presented is for All-IXE treatment period

*AEs* adverse events, *AESIs* adverse events of special interest, *CI* confidence interval, *IR* incidence rate, *IXE* ixekizumab, *MACE* major adverse cardiac events, *MedDRA* Medical Dictionary for Regulatory Activities, *N* population size, *n* number in group, *PsA* psoriatic arthritis, *PY* patient-years, *Q2W* every 2 weeks, *Q4W* every 4 weeks, *SAE* serious adverse event, *TEAE* treatment-emergent adverse event

**Fig. 3:**
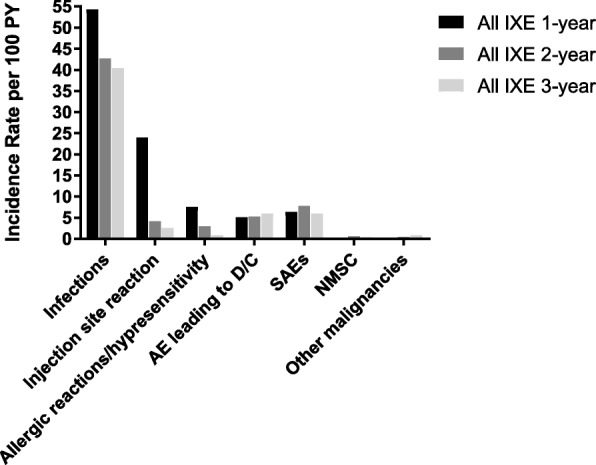
Treatment-emergent adverse events per 100 patient-years by years of treatment. AE: adverse event; D/C: discontinuation; IXE: ixekizumab; NMSC: non-melanoma skin cancer; PY: patient-years; SAE: serious adverse event

Likewise, the IRs for serious AEs (SAEs) remained stable with longer IXE treatment (Fig. 3). SAEs (*n* [IRs/100 PY]) occurring in ≥ 3 patients were cholelithiasis and pneumonia (5 [0.3/100 PY] each), bronchitis, and fall (4 [0.2/100 PY] each), coronary artery disease, meniscus injury, and osteoarthritis (3 [0.2/100 PY] each). Six deaths (0.3/100 PY) were reported (cerebrovascular accident, metastatic renal cell carcinoma, cardiorespiratory arrest, myocardial infarction, drowning, and pneumonia). None of these deaths were determined related to IXE treatment. TEAEs leading to IXE discontinuation (n [IRs/100 PY]) included latent TB (19 [1.0/100 PY]), ISR (3 [0.2/100 PY]), and pneumonia, myalgia, and cerebrovascular accident in which the exposure-adjusted IRs were 2 [0.1/100 PY] for each TEAE.

### Adverse events of special interest

**Fig. 4:**
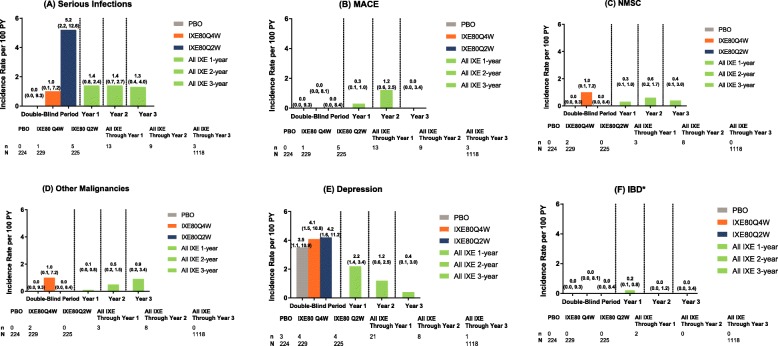
Exposure-adjusted incidence rate of TEAEs at 1-year intervals to year 3. The data points on the graph are the IR (95% CI)/100 PY at successive 1-year interval to year 3 for all ixekizumab-treated dataset (SPIRIT-P1, SPIRIT-P2, SPIRIT-P3) for **a** serious infections, **b** MACE (CEC-adjudicated), **c** NMSC, **d** other malignancies (excluding NMSC), **e** depression, and **f** IBD related. The CIs for the IRs are from likelihood ratio test of treatment effect from the Poisson regression model. The AEs were coded using MedDRA Version 19.1. ^*^95% CI was not evaluated for IBD. AE: adverse event; CEC: Clinical Events Committee; CI: confidence interval; IBD: inflammatory bowel disease; IR: incidence rate; IXE: ixekizumab; MACE: major adverse cardiovascular events; MedDRA: Medical Dictionary for Regulatory Activities; Ns: number of patients entered in each time interval; n: number in group; PBO: placebo; PY: patient-years; Q2W: every 2 weeks; Q4W: every 4 weeks; TEAEs: treatment-emergent adverse events

The IRs at 1-year intervals up to year 3 including double-blind treatment are shown in Fig. 4 for (a) serious infections, (b) MACE (CEC-adjudicated), (c) NMSC, (d) other malignancies (excluding NMSC), (e) depression, and (f) IBD related.

#### Infections

The IRs of infection-related TEAEs decreased with increasing duration of IXE exposure (Fig. 3). The most common infections (*n* [IRs/100 PY]) were upper respiratory tract infection (161 [8.8/100 PY]), nasopharyngitis (150 [8.2/100 PY]), and bronchitis (81 [4.4/100 PY]). The overall incidence of SI (*n* [IRs/100 PY]) was 23 patients (1.3/100PY). SIs (*n* [IRs/100 PY]) occurring in > 1 patient were pneumonia (5 [0.3/100 PY]), bronchitis (4 [0.2/100 PY]), and latent TB (hospitalization for testing to exclude active TB), lower respiratory tract infection, and esophageal candidiasis (2 [0.1/100 PY each]). The IRs for candida infections were 39 (2.1/100PY). No treatment-emergent candida infection resulted in IXE discontinuation.

There were 15 patients (0.8/100 PY) with localized herpes zoster. Twenty-one patients (1.2/100 PY) discontinued IXE because of infections: 6 patients (0.3/100 PY) due to latent TB, 2 patients (0.1/100 PY) due to pneumonia, and 1 patient (0.1/100 PY) each due to septic arthritis, bronchitis, cellulitis, dermatitis, folliculitis, hepatitis B, nasopharyngitis, otitis media, staphylococcal infection, subcutaneous abscess, tonsillitis, tooth abscess, and urinary tract infection. Grade 3 neutropenia (< 1000 cells/mm^3^ and ≥ 500 cells/mm^3^) occurred in 6 patients (0.3/100PY). The majority of cases of neutropenia were either Grade 2 (< 1500 cells/mm^3^ and ≥ 1000 cells/mm^3^) in 59 patients (3.2/100PY) or Grade 1 (<2000 cells/mm^3^ and ≥ 1500 cells/mm^3^) in 137 patients (7.5/100PY). No patients had infections temporally associated with neutropenia of Grade 3. The reported events were common types of non-opportunistic infections such as nasopharyngitis and otitis externa and influenza (1 patient each); none was a serious adverse event.

#### Injection-site reactions

The incidence of ISRs decreased substantially from the first year and remained stable over time (Fig. 3). The most common preferred terms of ISRs (*n* [IRs/100 PY]) were unspecified ISR (142 [7.8/100 PY]), injection-site erythema (52 [2.9/100 PY]), and injection-site pain (18 [1.0/100 PY]). There were 3.5 ISRs per 100 active injections. In most cases, ISRs did not result in treatment discontinuation, 6 patients (0.3/100 PY). There were no serious ISRs.

#### MACE

The incidence of MACE did not increase with longer IXE exposure (Fig. 3). Eleven patients (0.6/100 PY) had CEC-confirmed MACE (2 vascular deaths, 5 nonfatal myocardial infarctions, and 4 nonfatal strokes). Approximately 72% of the patients had one or more cardiovascular risk factors including hypertension, dyslipidemia, diabetes, and pre-existing cardiovascular disease.

#### Malignancy

With longer IXE exposure, there was no increase in the malignancy rate (Fig. 3). Thirteen patients (0.7/100 PY) developed malignancy. Of these, 8 patients had NMSC and 6 patients had breast cancer (*n* = 1), prostate cancer (*n* = 1), invasive ductal breast carcinoma (*n* = 1), malignant melanoma in situ (*n* = 1), metastatic renal cell carcinoma (*n* = 1), and papillary thyroid cancer (*n* = 1). These events were considered SAEs and led to discontinuation of study drug.

#### Hypersensitivity events

The IRs of hypersensitivity events decreased with increasing durations of IXE exposure (Fig. 3). There was one case of SAE of angioedema (non-anaphylactic reactions) and no case of anaphylaxis. Eight patients discontinued due to hypersensitivity including drug eruption, angioedema, dermatitis infected, injection-related reaction, rash, rash pruritic, and solar urticaria.

#### Inflammatory bowel disease

Two patients (IR = 0.1/100 PY; 1 CD, 1 UC) had adjudicated IBD, and these two patients did not have reported IBD history. Both of these events occurred at 6 months to 1 year of treatment with IXE Q2W group. Three patients (IR = 0.2/100 PY; 1 CD, 2 UC) had un-adjudicated IBD.

#### Other adverse events of special interest

There was no evidence of an increase in depression-related events over time (Fig. 3). The incidence of depression-related events were 1.6/100 PY. One patient (0.1/100PY) had a SAE of depression. Another patient discontinued due to a depression event; this patient was on IXE treatment and had a prior history of depression. The event was not considered related to the study drug. No suicide or self-injury-related behaviors were reported. One patient met laboratory criteria for potential drug-induced liver injury: a 59-year-old male who had received first dose of IXE during the blinded treatment period and was diagnosed with cholelithiasis approximately 2 years after starting the study. The patient underwent surgery and recovered; the event was considered not related to IXE.

## Discussion

Here, we report data from the IXE PsA program that includes 3 studies and 1822.2 PY of exposure. The overall TEAEs decreased or remained stable with longer IXE exposure. Consistent with previous reports, ISRs and upper respiratory tract infections are reported as the most frequent TEAEs [7]; the IR for these events decreased with an increase in the duration of IXE exposure. This is consistent with the pattern observed in the psoriasis clinical trials and with the findings associated with the use of secukinumab in PsA [9, 10]. This is similar to the reports for biologic agents neutralizing TNF [11].

The overall incidence of SIs was low which is consistent with this class of biologics [12–14]. Due to the impact on the immune-mediated natural defense, anti-TNF-α, a pro-inflammatory cytokine has been associated with an increased risk of infection, particularly reactivation of latent TB and fungal infections [15]. Results from the British Society for Rheumatology Biologics Register have reported non-significant increase in the rate of SIs between TNF-treated and control [16]. The German and Swedish Biologics Registries have reported a small but significant increase in the risk of SIs [17]. Similarly, for the Italian GISEA registry, the overall incidence of SIs was 31.8/1000 PY in a long-term treatment with anti-TNF therapy [18].

Patients with latent TB were allowed into the clinical trials if treatment was completed per the standard guidelines or were ongoing at the time of study inception; 32 (1.8/100PY) had treatment-emergent latent TB infection. There were no cases of TB reactivation or active TB in the PsA clinical program [7]. Several analyses, primarily from the European registries for biologics, have reported the association between TNF-α inhibitor administration and risk of TB infections; this is particularly true for anti-TNF monoclonal antibodies such as infliximab and ADA when compared with etanercept [19–21].

Consistent with the known mechanism of action of IXE and the role of IL-17 signaling in mucocutaneous defense, candida infections were the most common opportunistic infections [22]. The IRs/100 PY of candida infections and esophageal candidiasis in the present data from PsA.

were 2.1 and 0.1, respectively; most were mild or moderate in nature and there was no discontinuation due to candida infections. Consistent with reports from the psoriasis program and with studies on other IL-17 inhibitors, there were no deep organ or blood stream fungal infections [9, 12].

The role of IL-17 in the pathogenesis of IBD has not been clearly delineated, and patients with PsA have an increased risk for IBD compared with the background population [23, 24]. The IR for IBD for IXE remained consistent with background rates with 2 patients (0.1/100 PY) adjudicated with IBD; one each for CD and UC, respectively, both cases were new onset. Reports from other IL-17 inhibitors such as secukinumab have reported 3 cases of UC, 3 cases of CD and 2 cases of IBD unclassified (EAIRs 0.08, 0.08, and 0.05); 7 of these represented new onset cases [25].

Patients with PsA have an increased risk of MACE, and subjects at entry into the IXE PsA program had a prevalence of known cardiovascular risk factors of obesity (body mass index > 30) of 479 (42.8%), diabetes 78 (7.0%), dyslipidemia 30 (2.7%), and hypertension 434 (38.8%). The IR of CEC-confirmed MACE was 0.6/100 PY, with no trend for an increase with increasing IXE exposure. These findings are consistent with that reported in a pooled safety analysis of IXE from 3 clinical trials (0.7/100PY) [7].

Though severe psoriasis has been associated with increased risk of self-harm and suicide attempts relative to the general population (incidence rate ratios = 1.69), the literature in patients with PsA has been limited [26]. In compliance with the ICH guidelines, only patients with significant uncontrolled neuropsychiatric disorders were excluded, thus patients with a wide spectrum of stable neuropsychiatric disorders including depression were allowed into the ixekizumab PsA clinical trials. One serious depression-related event was reported; there was no suicide ideation, behavior, or completed suicide in the IXE PsA program. These findings are consistent with reports from other IL-17 inhibitors [27].

There were six deaths reported, with the causes being cerebrovascular accident, metastatic renal cell carcinoma, cardiopulmonary arrest, myocardial infarction, drowning, and pneumonia. Upon medical review, no deaths were attributed to IXE by the sponsor. These findings are consistent with previous reports in the larger psoriasis IXE-treated population [9].

Although this study covers up to 1822.2 PYs of exposure with IXE in patients with PsA, the duration of the program and the small number of AEs limit the conclusions that can be drawn for rare events or events. Due to limitations in the clinical trial setting including limited follow-up time with IXE exposure, ongoing long-term studies and post-marketing data will provide additional data to delineate the safety profile of IXE in this treatment population.

## Conclusions

The data presented in this report indicate a consistent safety profile for IXE over a period of 3 years. Additionally, the safety profile reported in the PsA treatment population remains consistent with the larger IXE psoriasis clinical trial program [9].

## Abbreviations

ADA: Adalimumab 40 mg
AEs: Adverse events
bDMARDs: Biologic disease-modifying antirheumatic drugs
cDMARDs: Conventional disease-modifying antirheumatic drugs
CEC: Clinical Events Committee
CD: Crohn’s disease
DMARDs: Disease-modifying antirheumatic drugs
IBD: Inflammatory bowel disease
ICH: International Council for Harmonization of Technical Requirements for Registration of Pharmaceuticals for Human Use
IL-17A: Interleukin 17A
IRs: Incidence rates
ISRs: Injection-site reactions
IXE: Ixekizumab
MACE: Major adverse cardiovascular event
MedDRA: Medical Dictionary for Regulatory Activities
NMSC: Non-melanoma skin cancer
PBO: Placebo
PsA: Psoriatic arthritis
PY: Patient-years
Q2W: Every 2 weeks
Q4W: Every 4 weeks
SAEs: Serious adverse events
SD: Standard deviation
SIs: Serious infections
TB: Tuberculosis
TEAE: Treatment-emergent adverse event
TNF: Tumor necrosis factor
TNF-α: Tumor necrosis factor alpha
UC: Ulcerative colitis
